# A Phase I clinical study of cisplatin-incorporated polymeric micelles (NC-6004) in patients with solid tumours

**DOI:** 10.1038/bjc.2011.6

**Published:** 2011-02-01

**Authors:** R Plummer, R H Wilson, H Calvert, A V Boddy, M Griffin, J Sludden, M J Tilby, M Eatock, D G Pearson, C J Ottley, Y Matsumura, K Kataoka, T Nishiya

**Affiliations:** 1 Northern Institute for Cancer Research, Paul O’Gorman Building, Framlington Place, Newcastle upon Tyne NE2 4AD, UK; 2 Centre for Cancer Research and Cell Biology, Queen's University Belfast, Lisburn Road, Belfast BT9 7AB, Northern Ireland, UK; 3 Department of Earth Sciences, University of Durham, Science Labs, Durham DH1 3LE, UK; 4 Investigative Treatment Division, Research Center for Innovative Oncology, National Cancer Center Hospital East, 6-5-1 Kashiwanoha, Kashiwa, Chiba 277-8577, Japan; 5 Department of Materials Engineering, Graduate School of Engineering, and Center for Disease Biology and Integrative Medicine, Graduate School of Medicine, The University of Tokyo, 7-3-1 Hongo, Bunkyo-ku, Tokyo 113-8656, Japan; 6 Division of Clinical Study, NanoCarrier Co. Ltd, Yaesu Yamagata Building, 3-2-2 Nihonbashi, Chuo-ku, Tokyo 103-0027, Japan

**Keywords:** cisplatin, DDS, EPR effect, NC-6004, phase I study, polymer micelle

## Abstract

**Background:**

On the basis of preclinical studies of NC-6004, a cisplatin-incorporated micellar formulation, we hypothesised that NC-6004 could show lower toxicity than cisplatin and show greater anti-tumour activity in phase I study.

**Methods:**

A total of 17 patients were recruited in a range of advanced solid tumour types. NC-6004 was administered intravenously (i.v.) every 3 weeks. The dose escalation started at 10 mg m^−2^ and was increased up to 120 mg m^−2^ according to the accelerated titration method and modified Fibonacci method.

**Results:**

One dose-limiting toxicity (DLT) occurred in a patient who was given 90 mg m^−2^ of NC-6004, otherwise any significant cisplatin-related toxicity was not observed or generally mild toxicity was observed. Despite the implementation of post-hydration and pre-medication regimen, renal impairment and hypersensitivity reactions still developed at 120 mg m^−2^, which led to the conclusion that the maximum tolerated dose was 120 mg m^−2^, and the recommended dose was 90 mg m^−2^, although DLT was not defined as per protocol. Stable disease was observed in seven patients. The maximum concentration and area under the concentration–time curve of ultrafilterable platinum at 120 mg m^−2^ NC-6004 were 34-fold smaller and 8.5-fold larger, respectively, than those for cisplatin.

**Conclusion:**

The delayed and sustained release of cisplatin after i.v. administration contributes to the low toxicity of NC-6004.

Cisplatin, *cis*-diamminedichloroplatinum (II), is a platinum (Pt)-based chemotherapy drug used to treat various types of cancers. Clinical use of cisplatin is, however, associated with irreversible renal toxicity, which necessitates the use of pre- and post-hydration regimens, and excludes its use in patients with less than normal renal function (Pinzani *et al*, 1994). Cisplatin therapy also causes neurotoxicity, gastrointestinal toxicity (nausea and vomiting), haematological toxicity, and irreversible ototoxicity (Hartmann and Lipp, 2003). Furthermore, its anti-tumour efficacy continues to be limited by either intrinsic or acquired resistance (Kartalou and Essigmann, 2001). To overcome these cisplatin-related disadvantages, various types of Pt analogues, including carboplatin, oxaliplatin, satraplatin, and picoplatin have been developed (Kelland and Sharp, 1999; Judson and Kelland, 2000; Sharp *et al*, 2002). Another potential method for improving the therapeutic indices of cisplatin is the incorporation of cisplatin into polymeric micelles of varying size in the range of 20–100 nm composed of polyethylene glycol (PEG)-poly (amino acid) block co-polymers, in which PEG constitutes the hydrophilic outer shell of the micelle and cisplatin is incorporated into hydrophobic inner core of the micelle (Yokoyama *et al*, 1996; Nishiyama *et al*, 1999, 2001b, 2003; Nishiyama and Kataoka, 2001a).

Preclinical studies carried out on NC-6004, cisplatin-incorporated polymeric micelles composed of PEG-poly (glutamic acid) block co-polymers via polymer–metal complex formation (Figures 1 and 2), have indicated that it is preferentially distributed to tumours by enhanced permeability and retention effect (Matsumura and Maeda, 1986; Maeda and Matsumura, 1989; Maeda *et al*, 2000; Maeda, 2001), and demonstrates significantly lower toxicity than cisplatin and greater anti-tumour activity (Uchino *et al*, 2005). On the basis of these results, a phase I clinical trial of NC-6004 in patients with advanced solid tumours has been carried out. The objectives of the study were to determine the maximum-tolerated dose (MTD), the recommended dose (RD) for the phase II, the dose-limiting toxicities (DLTs), the safety and tolerability profile, and to explore evidence of anti-tumour activity, and the pharmacokinetics of NC-6004.

## Patients and methods

### Ethics

The trial was an open-label, dose-escalating, phase I study conducted at two sites in the United Kingdom; Newcastle General Hospital and Belfast City Hospital. All procedures were reviewed by Independent Ethics Committees, and were in accordance with the protocol, the Helsinki Declaration (October 2000, and clarified 2002 and 2004), the Note for Guidance on Good Clinical Practice (CPMP/ICH/135/95) approved in July 1996, and the applicable regulatory requirements.

### Administration of therapeutic agent

NC-6004 (NanoCarrier Co., Ltd Chiba, Japan) was a sterile solution containing the equivalent of 2.5 mg ml^−1^ cisplatin and could be diluted in 5% dextrose before administration.

Dosing was performed by intravenous (i.v.) infusion of 500 ml over 60 min, once every 3 weeks. Following the observation of renal toxicities, hydration using 1000 to 1500 ml of fluid, immediately after NC-6004 infusion, was implemented for the rest of the study. Later in the study, after the occurrence of four events of hypersensitivity reactions, the following prophylactic treatment was implemented at each cycle for all patients; 30 min before infusion – dexamethasone 20 mg i.v., chlorphenamine 10 mg i.v., and ranitidine 50 mg i.v. Oral dexamethasone 4 mg (twice a day), ranitidine 150 mg (twice a day), and chlorphenamine 4 mg (three times a day) could also be given if necessary on a per-patient individualised basis for 48 h after infusion.

### Patients’ eligibility and dose escalation

Patients with histologically confirmed advanced solid tumours, for which no standard therapy exists or has failed therapy, were eligible for enrolment in this study, provided that the following criteria were met: Eastern Cooperative Oncology Group performance status of ⩽2; age of ⩾18 years; life expectancy of at least 12 weeks; a normal haematological profile, renal function, hepatic function, and serum calcium level, no more than one previous course of Pt therapy, with maximum cumulative doses of 480 mg m^−2^ of cisplatin, 1040 mg m^−2^ of oxaliplatin, or 42 mg ml^−1^ min^−1^ (min=minutes) cumulative area under the concentration-time curve (AUC) of carboplatin, and no chemotherapy, no radiotherapy (except palliative radiation delivered to <20% of bone marrow), no immunotherapy, or no corticosteroids (greater than 10 mg per day of prednisone or equivalent) within 4 weeks before entering the study or patients who have not recovered from adverse events because of agents administered more than 4 weeks earlier. Patients who had severe hypersensitivity to Pt compounds, ototoxicity assessed by audiometry (except senile hearing loss at high frequency) or other neurotoxicity ⩾ grade 2 were ineligible for enrolment in the study. Patients were excluded if they were pregnant or lactating.

The dose of NC-6004 is, hereafter, always expressed as cisplatin equivalent mg m^−2^ of body surface area per injection. The starting dose of NC-6004 was 10 mg m^−2^, one-tenth of the lethal dose in 10% in rat or one-third of the toxic dose low in dog. In stage 1, (accelerated titration method), each dose was escalated at twice the previous dose level until drug-related toxicity ⩾grade 2 was seen in cycle 1. Once the first drug-related toxicity ⩾grade 2 in cycle 1 was seen, a minimum cohort of three patients was recruited, and each dose was defined as 150% of the previous one in stage 2 (modified Fibonacci method) until MTD was reached. The dose was modified according to the estimated creatinine clearance (Cl)/GFR measured before each administration of NC-6004 as detailed in Table 1. Intra-patient dose escalation was not permitted.

Toxicity was graded by the Common Terminology Criteria for Adverse Events version 3.0. MTD was defined as the dose at which one-third of patients experience DLT, and RD was the highest dose, which gave rise to no more than one DLT out of a cohort of six patients. DLTs were defined as grade 4 neutropenia associated with fever (⩾38.5°C) or diarrhoea ⩾ grade 2, grade 4 neutropenia lasting ⩾5 days without fever, grade 4 thrombocytopenia for ⩾5 days, grade 3 or higher non-haematological toxicity (except liver transaminase elevation, or nausea or vomiting treatable by anti-emetic), and treatment delay >2 weeks before start of next cycle of treatment because of unresolved toxicity.

### Pretreatment assessment and follow-up studies

Assessment of medical history was completed during the 21 days before the start of NC-6004 dosing. Safety was monitored throughout the trial until the end of trial visit. In the treatment phase, physical assessment, routine laboratory analysis, estimated creatinine Cl, and concurrent illness/therapy were reviewed on day 1 of cycle 1, then every week until week 7 and then every 3 weeks thereafter, and at withdrawal from the trial. Adverse events were reviewed during the first 4 days of cycle 1, then every week from weeks 2 to 7 and then every 3 weeks from week 7 onwards, and at withdrawal. The CT/MRI scans of all target and non-target lesions were performed every 6 weeks and at withdrawal, and tumour markers, if applicable, were assessed every 3 weeks. The Response Evaluation Criteria in Solid Tumour (RECIST) was used to define lesions and the criteria for objective tumour response. For pharmacokinetic analysis, blood samples were taken at 0, 2, 4 and 8 h after administration on days 1, 2, 3, 4, 8, 15, and 22 and before cycle 2.

### Pharmacokinetic analysis

Blood samples were centrifuged and separated plasma was processed to produce three different forms of sample: total plasma, gel filtrate and ultrafiltrate. Plasma (1 ml) was stored for total-plasma Pt analysis. Plasma (1 ml) was centrifuged with a molecular weight cutoff of 200 000 Da, and the eluant was analysed for micellar Pt. Finally, a further 1 ml of plasma was centrifuged with a molecular weight cutoff of 30 000 Da to give an ultrafiltrate for the determination of low-molecular weight Pt species, including cisplatin. Concentration of total plasma Pt and micellar Pt (gel filtrate) were measured using atomic absorption spectrometry on Analyst 600 (Perkin-Elmer, Waltham, MA, USA) against standards prepared in plasma. Ultrafiltrate samples were analysed by inductively coupled plasma mass spectrometry on Element 2 (Thermo Scientific Inc., Waltham, MA, USA) against centrifuged Pt standards at Durham University. Pharmacokinetic analysis was performed using WinNonlin version 1.3 (Pharsight Corporation, Mountain View, CA, USA) to calculate the maximum concentration (*C*_max_), the time to the maximum concentration (*T*_max_), elimination half-life (*t*_1/2_), and the AUC from zero to infinity (AUC_inf_) for all Pt species. Clearance (Cl) and volume of distribution (*V*_z_) were calculated for total plasma Pt.

## Results

### Patient characteristics

The first patient was dosed on 15 May 2006 and the last study exit visit occurred on 6 February 2008. In total, 17 patients were enrolled and each received at least one dose of NC-6004, representing the intention-to-treat population. Demographic characteristics of patients are summarised in Table 2. All recruited patients were Caucasian, with a median height of 170.0 cm and a median weight of 73.0 kg. Cancer history of patients is summarised in Table 3. The range of tumour types was large, with no specific tumour type represented more across the different groups. Tumour stage was similar between the dosing cohorts.

### Dosing and toxicity

The process for dose escalation is shown in Table 3. In total, 41 doses were administered to 17 patients. The maximum number of treatments was four cycles in three patients, and the mean number of administrations per patient was 2.4 cycles. Dose escalation started at 10 mg m^−2^ and was increased up to 40 mg m^−2^ following the accelerated titration method. Owing to grade 2 renal toxicity in cycle 1 of a patient at 40 mg m^−2^, reported as a serious adverse event (SAE), the study entered stage 2 with a dose escalation up to 120 mg m^−2^ according to modified Fibonacci method.

Infusion-related adverse events are summarised in Table 4. NC-6004 injection was well tolerated in terms of haematological toxicities. Thus, one episode of grade 3 thrombocytopenia at 10 mg m^−2^ and grade 1 thrombocytopenia at 90 mg m^−2^ only were observed (not DLTs). For non-haematological toxicity, the most frequent related adverse events were fatigue (52.9%), anorexia and nausea (47.1%), vomiting (41.2%), and hypersensitivity reaction and renal impairment (35.3%). Significant cisplatin-related ototoxicity and neurotoxicity were not observed at any dose level. One out of six patients at 90 mg m^−2^ experienced grade 3 fatigue in cycle 1 (DLT). One out of three patients at 60 mg m^−2^ had grade 3 vomiting in cycle 1, and one patient in each 60, 90, and 120 mg m^−2^ developed grade 3 hypersensitivity reaction (not DLTs). The clinical signs and symptoms of hypersensitivity reactions to NC-6004 were urticarial rash, dizziness, sweating, cough, dyspnoea, hypotension, tingling, swelling of tongue, lip, and pharynx, tightness in chest, and burning sensation, some of which are typical reactions for Pt, and they always developed after a minimum of two cycles of NC-6004. Other infusion-related toxicities were grade 2 or lower. Despite the implementation of post hydration (from 40 mg m^−2^ onwards) and hypersensitivity prophylaxis (from 90 mg m^−2^ onwards), grade 2 renal toxicity accompanied by a reduction in dose and/or delay in dose for 1 week was still observed at 90 and 120 mg m^−2^, and grade 2 and 3 hypersensitivity reactions (SAEs) also developed at 120 mg m^−2^. Following these events, it was considered that adding further patients or increasing the dose level would not be reasonable, and the study was discontinued at dosage level of 120 mg m^−2^. As the effect on renal function at 90 mg m^−2^ dosage was less marked than that observed at 120 mg m^−2^, the 120 mg m^−2^ dosage was considered to be the MTD, and the RD of NC-6004 as monotherapy for further studies was therefore estimated to be 90 mg m^−2^, although renal toxicity and hypersensitivity reactions were not defined as potential DLT per protocol.

### Therapeutic response

Best overall response calculated by RECIST is shown in Table 3. No patient was assessed as complete response or partial response. Seven patients (41.2%) were evaluated as having had a stable disease (SD) for longer than 4 weeks at the time of the study completion, even though six of these had advanced Stage IV solid tumours. It should be noted that only two out of eight patients (25%) at the dose levels from 10 to 60 mg m^−2^ had a best response of SD, however the SD ratios at 90 and 120 mg m^−2^ were 50 and 67% respectively, suggesting that the efficacy of NC-6004 is more pronounced at higher dose levels. Overall, 14 patients (82.4%) died or experienced tumour progression, and median progression-free survival time was 49 days.

### Pharmacokinetics

**Figure 3:**
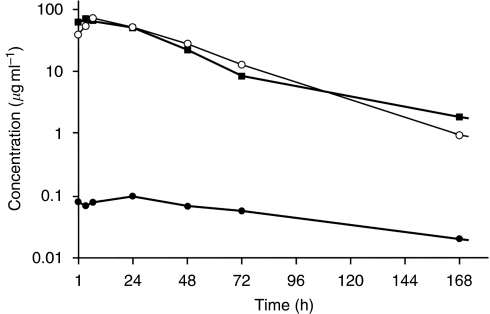
Plasma Pt concentration–time curve from a patient treated at 90 mg m^−2^ of NC-6004. ▪, total Pt, ○, gel-filterable Pt, •, ultrafilterable Pt.

Pharmacokinetic parameters for Pt measured per cohort in the three different matrices are shown in Table 5. A typical plasma concentration–time profile is also shown in Figure 3.

Pharmacokinetics of total plasma Pt was characterised by longer *t*_1/2_, and higher *C*_max_ and AUC_inf_ with smaller *V*_z_ and Cl compared with those of cisplatin, indicating that the blood circulation of cisplatin was prolonged by the incorporation into the micelles. Thus, the *C*_max_ and AUC_inf_ of NC-6004 at 120 mg m^−2^ were approximately 11-fold higher than those of cisplatin at an equivalent dose (Kitajima *et al*, 1987). The *t*_1/2_ of NC-6004 at 120 mg m^−2^ was longer than that of cisplatin at an equivalent dose, 14.6 min for the initial phase and 73.8 h for the terminal phase (Kitajima *et al*, 1987). The *V*_z_ and Cl of NC-6004 were smaller than those of cisplatin, 52 l and 350 ml h^−1^, respectively (Calvert *et al*, 1993). The AUC_inf_ and *C*_max_ of NC-6004 increased in a dose-dependent manner, and there was no apparent change in Cl with increasing dose.

For the gel-filterable Pt (intact micellar formulation), the *T*_max_ was similar to that of total plasma Pt, the *t*_1/2_ generally mirrored that of total plasma Pt, and the *C*_max_ and AUC_inf_ values were approximately 88% of those of total plasma Pt.

For the ultrafilterable Pt (active species including cisplatin), the *C*_max_ at 120 mg m^−2^ was 34-fold lower than that of non-protein-bound cisplatin after the administration of an equivalent dose of cisplatin (Kitajima *et al*, 1987), which might be responsible for the lower incidence of toxicity compared with that associated with cisplatin therapy. Conversely, *t*_*1*/2_ and AUC_inf_ at 120 mg m^−2^ of NC-6004 were 230-fold and 8.5-fold larger, respectively, than those of non-protein-bound cisplatin after the administration of an equivalent dose of cisplatin (Kitajima *et al*, 1987). The persistence of active Pt species might indicate an improved efficacy of NC-6004. Furthermore, *T*_max_ (24 h or greater) was delayed compared with that of total plasma Pt or gel-filterable Pt, suggesting that NC-6004 provides a delayed and sustained release of potentially active Pt species after the administration period.

## Discussion

NC-6004 was well tolerated with minimal nephrotoxicity and no significant myelosuppression, ototoxicity, emesis, or neurotoxicity, but a higher rate of hypersensitivity reactions than predicted. No DLT per protocol was seen at doses up to 90 mg m^−2^ where 1 DLT (grade 3 fatigue) was experienced by one out of six patients, and no further DLT per protocol was seen at 120 mg m^−2^ when the study was discontinued. In general, the toxicities of NC-6004 were less severe and less frequent compared with cisplatin, particularly nausea/vomiting, anorexia, alopecia, and haematological toxicity.

In this study, dose delays/reductions were mainly due to effects on renal function. Despite the introduction of 1 to 1.5 l of fluid over 2 h, following NC-6004 administration, rising creatinine and/or reduction in estimated creatinine Cl or ^51^Cr-EDTA Cl affected two out of six patients at 90 mg m^−2^ and two out of three patients at 120 mg m^−2^, although the creatinine level returned to baseline in 2 weeks. Cisplatin therapy requires a total 8 h hydration, comprising 1–2 l over 4 h of hydration, both before and after the administration of cisplatin, to prevent nephrotoxicity. Another potential advantage of NC-6004 over cisplatin is, therefore, the reduced need for hydration, and that renal impairment was kept to minimum by modest hydration. Whether hydration is absolutely necessary for NC-6004 therapy to reduce the incidence of renal impairment remains to be assessed in a future trial.

Dose interruptions due to toxicity in this study were all related to hypersensitivity reactions, which occurred unpredictably at four out of six dose levels (10, 60, 90, and 120 mg m^−2^) in six patients. The first three patients had previous Pt therapy and the last three patients were Pt-naïve, thus, the occurrence of hypersensitivity reaction depends on neither dose level nor the previous Pt exposure. The use of a prophylactic regimen of dexamethasone, ranitidine, and chlorphenamine, previously described, was not sufficient to prevent hypersensitivity reactions in two patients at 120 mg m^−2^, therefore a more stringent prophylactic regimen (Kwon *et al*, 2002) might be necessary. As most of the patients recruited in this Phase I study progressed by the end of cycle 2, and hypersensitivity reactions developed after a minimum of two cycles of NC-6004, despite the pre-medication, it was considered that this phase I study was not the appropriate setting to assess alternative pre-medication strategies. Therefore, the study was discontinued, so that this problem could be assessed in a future trial. In preclinical studies, the antigenicity of NC-6004 was examined compared with cisplatin, polymer vehicle, polymer-bound cisplatin (not in a micelle form), and cisplatin–plasma protein complex. The results indicated that cisplatin and polymer vehicle are not antigenic, and the highest extent of antigenicity observed was in cisplatin–plasma protein complex, followed by NC-6004 and then polymer-bound cisplatin (not in a micelle form). This suggests that the hypersensitivity reaction to NC-6004 may have been due to plasma protein-bound cisplatin, which is formed by rapid binding of plasma protein to released cisplatin, which then circulates in the blood for a prolonged period. However, the mechanism has not yet been fully clarified.

Taking account of the incidence of hypersensitivity reaction and renal impairment, 120 mg m^−2^ was considered to be close to the MTD, such that 90 mg m^−2^ was most likely the RD for monotherapy for future studies, although the definition per protocol of the MTD was not actually reached.

In spite of the patients generally being heavily pretreated, some evidence of disease stabilisation was seen, and seven patients demonstrated SD after 6 weeks of treatment. Efficacy will be further assessed in a future trial.

The pharmacokinetic analysis indicated the prolonged circulation of NC-6004 in the blood, and delayed and sustained release of potentially active Pt species after the administration of NC-6004. More importantly, the observed lower *C*_max_ for ultrafilterable Pt compared with that of non-protein-bound cisplatin, after the cisplatin injection, might result in the reduction of cisplatin-related toxicity. Furthermore, the higher AUC_inf_ and *t*_1/2_ for ultrafilterable Pt compared with that of non-protein-bound cisplatin after the cisplatin injection might enhance the efficacy of NC-6004. However, an increase in the AUC of plasma protein-bound cisplatin because of rapid binding of plasma protein to released cisplatin might result in a higher risk of hypersensitivity reaction.

In conclusion, this Phase I study has confirmed that NC-6004 exhibits pharmacokinetic characteristics completely different from those of cisplatin, resulting in the reduction of cisplatin-related toxicity and the improvement of patient's quality of life so that the patients can take therapy without hospitalisation for hydration and treatment of cisplatin-related toxicities. The data obtained from this study are believed to open new avenues for the use of this micellar formulation in the clinic. The assessment of the most appropriate prophylactic regimen for hypersensitivity reactions, whether hydration is necessary and of efficacy are now underway in ongoing NC-6004 studies.

## Figures and Tables

**Figure 1:**
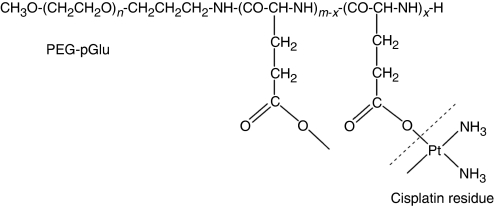
Structure of cisplatin-PEG-poly(glutamic acid) block co-polymer conjugate. PEG-pGlu, PEG-poly(glutamic acid); *n*, approximately 268; *m*, approximately 40; *x*, approximately 24.

**Figure 2:**
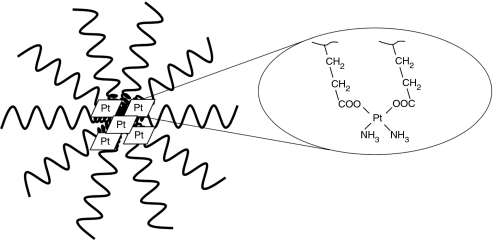
Structure of cisplatin-incorporated polymeric micelle, NC-6004. Core part, cisplatin residue bound to poly-L-glutamic acid. Exterior part, PEG.

**Table 1:** Dose modification for changes in estimated creatinine clearance or ^51^Cr-EDTA clearance

**Estimated creatinine clearance/GFR**	**NC-6004 dose**	**^51^Cr-EDTA clearance (in ml min^−1^)**	**NC-6004 dose**
>60 ml min^−1^	100% of dose	>60	100% of dose
50–60 ml min^−1^	80% of dose	40–60	50% of dose
<50 ml min^−1^ or any drop from	^51^Cr-EDTA clearance measurement	<40	Discontinue
baseline by >10% calculated GFR			

Abbreviations: ^51^Cr-EDTA clearance=chromium-51-ethylenediaminetetraacetic acid clearance; GFR=glomerular filtration rate.

**Table 2:** Patient characteristics

	**NC-6004 dose level (in mg m^−2^**)						
	**10**	**20**	**40**	**60**	**90**	**120**	**Total**
***n***	**1**	**1**	**3**	**3**	**6**	**3**	**17**
*Age (years)*							
Range	55	63	45–65	45–56	48–80	40–71	40–80
							
*Sex*							
Male	1	0	2	2	4	1	10
Female	0	1	1	1	2	2	7
							
*ECOG PS* a							
0	1	0	2	1	4	2	10
1	0	0	1	0	1	1	3
2	0	1	0	1	1	0	3
							
*Previous treatment*							
Chemotherapy	1	1	3	3	5	2	15
Surgery	0	0	3	3	3	3	12
Radiotherapy	0	1	1	2	0	3	7
Other therapies for cancer (targeted therapy, immunotherapy, or epigenetic therapy)	0	0	0	2	1	2	5

Abbreviation: ECOG PS=Eastern Cooperative Oncology Group performance status.

a For one patient at 60 mg m^−2^, ECOG PS was not assessed at screening, but was assessed at day 1 before infusion.

**Table 3:** Process for dose escalation

**Dose (mg m^−2^)**	**Patient no.**	**Primary tumour (stage)**	**Cycles received**	**No. of DLT**	**Events**	**Best overall response**
*Stage 1*						
10	101	Lung (IV)	3	0	Hypersensitivity reaction at cycle 3 (previous cisplatin therapy)	SD
20	102	Lung (IV)	2	0		PD
40	103	Colon (IV)	1	0	Grade 2 reduced renal function at cycle 1 (SAE). Cohort was expanded with two more patients.	PD
	204	Hepatic cell (IV)	4	0		SD
	105	Colon (IV)	2	0	Grade 1 reduced renal function at cycle 1. Hydration was implemented for the rest of study.	PD
						
*Stage 2*						
60	106	Mesothelioma (IIIA)	2	0	Hypersensitivity reaction at cycle 2 (previous carboplatin therapy).	PD
	207	Colon (IV)	2	0	Hypersensitivity reaction at cycle 2 (previous oxaliplatin therapy).	PD
	108	Oesophagus (IV)	2	0		NE
90	209	Pancreas (IIA)	4	0	Hypersensitivity reaction at cycle 4 (Pt-naïve). Prophylactic treatment was implemented for the rest of study.	SD
	110	Oesophagus (IV)	2	0	Grade 2 reduced renal function at cycle 1.	PD
	112	GIST (IV)	2	0	Grade 2 reduced renal function at cycle 1. Cohort was expanded with three more patients.	PD
	113	Lung (IV)	2	0		SD
	114	Pancreas (IV)	2	1	Grade 3 fatigue at cycle 1 (DLT).	SD
	215	Colon (IV)	2	0		PD
120	216	Melanoma (IV)	2	0	Grade 2 reduced renal function at cycle 1.	PD
	117	Melanoma (IV)	4	0	Grade 2 reduced renal function at cycle 1. Hypersensitivity reaction at cycle 4 (SAE) (Pt-naïve).	SD
	218	Renal cell (IV)	3	0	Hypersensitivity reaction at cycle 3 (SAE) (Pt-naïve).	SD

Abbreviations: DLT=dose-limiting toxicity; GIST=gastrointestinal stromal tumour; PD=progressive disease; Pt=platinum; NE=not estimated; SAE=serious adverse event; SD=stable disease.

**Table 4:** Summary of all related adverse events

	**NC-6004 dose level (in mg m^−2^**)						
	**10**	**20**	**40**	**60**	**90**	**120**	**Total**
***n***	**1**	**1**	**3**	**3**	**6**	**3**	**17**
*Haematological toxicity*							
Blood and lymphatic system disorders							
Thrombocytopenia	1	0	0	0	1	0	2
							
*Non-haematological toxicity*							
Gastrointestinal disorder							
Constipation	0	0	0	0	0	1	1
Dry mouth	0	0	0	1	0	0	1
Nausea	0	1	1	1	4	1	8
Paraesthesia oral	0	0	0	0	0	1	1
Tongue ulceration	0	0	0	0	1	0	1
Vomiting	0	0	1	1	4	1	7
General disorder and administration site conditions							
Fatigue	1	1	0	1	4	2	9
Infusion site reaction	0	1	0	0	0	0	1
Malaise	0	0	0	0	0	1	1
Immune system disorders							
Hypersensitivity	1	0	0	2	1	2	6
Metabolism and nutrition disorders							
Anorexia	0	0	2	1	4	1	8
Decreased appetite	0	0	0	1	0	0	1
Dehydration	0	0	0	0	1	0	1
Hypomagnesemia	0	0	0	0	1	0	1
Nervous system disorder							
Dizziness	0	0	0	0	0	1	1
Neuropathy peripheral	1	0	0	1	0	0	2
Peripheral sensory neuropathy	0	0	0	0	1	0	1
Renal and urinary disorder							
Renal impairment	0	0	2	0	2	2	6
Skin and subcutaneous tissue disorder							
Alopecia	0	0	0	0	0	1	1
Rash	0	0	0	0	0	1	1

**Table 5:** Pharmacokinetic parameters per cohort for total, micellar, and ultrafiltrable Pt of NC-6004 (mean±s.d.)

**Analyte**	**Dose (mg m^−2^)**	***T*_max_ (h)**	***C*_max_ (*μ*g ml^−1^)**	***t*_1/2_ (h)**	**AUC_inf_ (h *μ*g ml^−1^)**	***V*_z_ (l)**	**Cl (ml h^−1^)**
Total Pt	10	4.0	5.70	24	234	3.0	85
	20	5.4	12.20	20	492	2.0	68
	40	2.0±0.1	25.9±2.4	62±18	1135±78	5.5±2.0	62±11
	60	2.7±1.2	29.9±13.8	93±41	1354±638	6.7±0.5	107±69
	90	5.2±2.2	60.8±12.5	129±40	2836±554	11.8±6.9	61±20
	120	4.4±2.5	85.4±10.8	158±48	4377±563	10.9±3.8	48±9
							
Micellar Pt	10	—a	—a	—a	—a		
	20	6.4	8.90	16	237		
	40	2.0±0.1	13.9±10.4	67±56	509±204		
	60	5.0±1.7	14.4±7.3	18±7	385±153		
	90	4.8±2.4	42.4±20.3	39±27	1579±939		
	120	3.1±1.5	84.6±8.1	87±37	3857±1171		
							
UF Pt	10	48.7	0.009	114	1.7		
	20	23.5	0.022	71	2.5		
	40	24.0±0.1	0.045±0.014	141±126	4.7±2.1		
	60	26.5±20.9	0.096±0.022	114±47	13.2±5.8		
	90^b^	20.5±7.6	0.205±0.114	123±44	22.6±10.0		
	120^c^	26.4	0.131	115	22.9		

Abbreviations: AUC_inf_=area under concentration–time curve from zero to infinity; *C*max=maximum concentration; Cl=clearance; Pt=platinum; QC=quality control; *T*_max_=time to maximum concentration; UF Pt=ultrafilterable platinum; *V*z=volume of distribution.

a Data not valid – QCs out with acceptance limit.

b 1 data was not valid – QCs out with acceptance limit.

c 2 data were not valid – QCs out with acceptance limit.
